# Wall Teichoic Acids Are Involved in the Medium-Induced Loss of Function of the Autolysin CD11 against *Clostridium difficile*

**DOI:** 10.1038/srep35616

**Published:** 2016-10-19

**Authors:** Xia Wu, Elena E. Paskaleva, Krunal K. Mehta, Jonathan S. Dordick, Ravi S. Kane

**Affiliations:** 1 Center for Biotechnology and Interdisciplinary Studies, Rensselaer Polytechnic Institute, Troy, NY, US; 2 Department of Chemical and Biological Engineering, Rensselaer Polytechnic Institute, Troy, NY, US; 3 School of Chemical & Biomolecular Engineering, Georgia Institute of Technology, Atlanta, GA, US

## Abstract

Bacterial lysins are potent antibacterial enzymes with potential applications in the treatment of bacterial infections. Some lysins lose activity in the growth media of target bacteria, and the underlying mechanism remains unclear. Here we use CD11, an autolysin of *Clostridium difficile*, as a model lysin to demonstrate that the inability of this enzyme to kill *C. difficile* in growth medium is not associated with inhibition of the enzyme activity by medium, or the modification of the cell wall peptidoglycan. Rather, wall teichoic acids (WTAs) appear to prevent the enzyme from binding to the cells and cleaving the cell wall peptidoglycan. By partially blocking the biosynthetic pathway of WTAs with tunicamycin, cell binding improved and the lytic efficacy of CD11 was significantly enhanced. This is the first report of the mechanism of lysin inactivation in growth medium, and provides insights into understanding the behavior of lysins in complex environments, including the gastrointestinal tract.

The past few decades have witnessed an increasing interest in bacterial lytic enzymes (also called lysins) as potent antimicrobial agents against pathogenic bacteria. Lysins cleave specific types of cell wall peptidoglycan and cause lysis and death of their target bacterial cells^1^,^2^. The major classes of lysins attracting the most attention include endolysins, autolysins, virion-associated lysins (VALs), and class IIIa bacteriocins (or bacteriolysins). Both VALs and endolysins are encoded by bacteriophages and are essential for host infection and progeny release, respectively^1^,^2^,^3^,^4^,^5^. Autolysins are endogenous cell wall hydrolases and play active roles in cell wall synthesis and remodeling, and cell division^6^,^7^,^8^. Bacteriolysins are generated and secreted by certain bacteria to selectively target other competing bacteria^9^,^10^,^11^.

Lysins have been shown to be highly specific and efficient in killing Gram-positive bacteria when applied externally^1^,^2^,^12^,^13^, and have shown potential applications in detection, disinfection, and therapy^12^,^14^. Lysins have been used to kill a wide variety of pathogens, many of which are antibiotic resistant, without triggering common resistance mechanisms in target bacteria^1^,^2^,^12^,^15^,^16^,^17^,^18^,^19^,^20^,^21^. Proença *et al*. have noted, however, that the high lytic activity observed in buffered solutions often does not translate to the expected results in animal infection models^3^. Moreover, while most studies have focused on the activities of lysins in various buffered conditions that do not support active cell growth, some lysins are less active or inactive in rich growth media^3^,^6^,^22^. For example, the chimeric lytic enzyme SsaALP-LBD retained only 19% of its native activity against *Staphylococcus aureus* SA113 in MHIIB medium compared with that in phosphate buffered saline (PBS), and was severely inhibited in 10% human serum^6^. Although lysostaphin was active in tryptic soy broth supplemented with 0.25% glucose, a 16-fold higher enzyme concentration was required than in PBS to achieve successful eradication of *S. aureus* ATCC 35556 in biofilms^22^. In another example, while the endolysin Lys170 displayed lytic activity against *Enterococcus faecalis* clinical strains when these were collected from exponentially growing cultures and resuspended in a physiologic buffer prior to enzyme addition, it exhibited very poor lytic activity when added directly to logarithmic phase cultures in rich media^3^,^23^.

Upon infection, pathogenic bacteria usually multiply quickly in a short period, indicating the abundance of nutrients to support cell growth and proliferation^3^,^6^. Thus, inactivation of lysins in metabolism-sustaining environments imposes a major challenge to therapeutic applications. In addition, the presence of diet-derived nutrients in the gastrointestinal tract, where many bacterial infections occur, makes it challenging to develop therapeutic lysins. As a result, it is important to study the activities and specificities of lysins under various conditions, especially complex environments containing growth-supporting nutrients.

*Clostridium difficile* is a Gram-positive, spore-forming, toxin-producing anaerobic bacterium that causes diarrhea and pseudomembranous colitis, and in some cases toxic megacolon, perforation, peritonitis, and death^24^,^25^,^26^. *C. difficile* is the most prevalent cause of nosocomial diarrhea in the world^27^,^28^, and its infection is usually caused by prior treatment with broad-spectrum antibiotics^26^. *C. difficile* is resistant to many antibiotics^29^, and can only be treated with metronidazole, vancomycin, or clindamycin^26^.

As opposed to conventional antibiotics, lysins have been shown to be active against *C. difficile in vitro*. For example, CD27L is a potent endolysin against *C. difficile* with zinc-dependent *N*-acetylmuramoyl-L-alanine amidase activity^30^,^31^. We previously identified an autolysin CD11 with similar activity to CD27L^32^. CD11 reduces the viability of mid-log phase *C. difficile* cells by 3–4 log units in 3 h in aqueous buffer and is active against a wide range of clinical isolates, making it attractive for *in vivo* therapeutic use^32^. Nevertheless, CD11 is almost completely inactive in *C. difficile* growth medium. To elucidate the mechanism(s) of this medium-induced loss of CD11 activity, we investigated the lytic activity and substrate binding capacity of CD11 on intact cells and isolated cell wall materials in the presence of individual medium components. Our results suggest that a key reason for the dysfunction of CD11 in *C. difficile* growth medium is the inaccessibility of the cell wall to this enzyme. Wall teichoic acids (WTAs) appear to control access of the enzyme to the cell wall under different nutrient conditions, and hence, significantly contribute to the lack of enzyme binding to and lytic activity against *C. difficile* in growth medium. Our work provides important guidance to gaining a basic understanding of lysin function, and the development of lysin-based antimicrobial agents for applications in complex environments.

## Results

### Effect of medium components on enzyme activity on live cells and isolated cell wall

**Figure 1:**
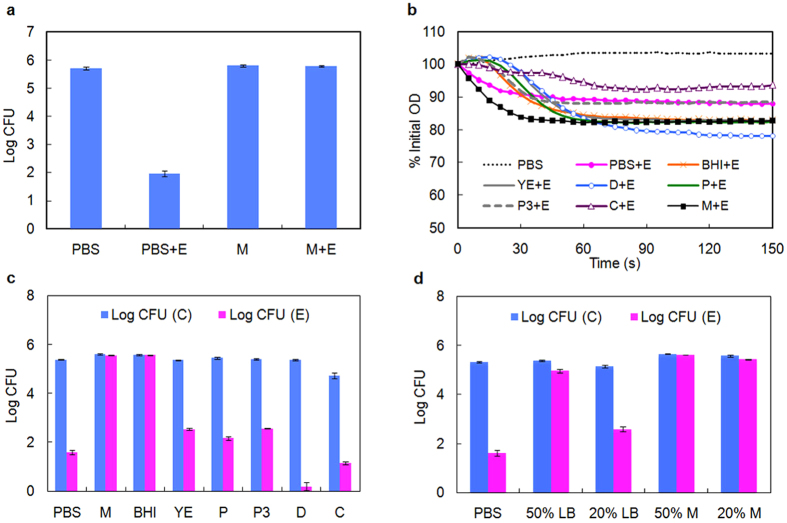
Activity of CD11 under different nutrient conditions. (**a**) Activity of CD11 against *C. difficile* cells in buffer and growth medium. (**b**) Activity of CD11 against isolated *C. difficile* cell wall fragments. (**c**) Activity of CD11 against mid-log phase *C. difficile* cells in medium components. (**d**) Activity of CD11 against *C. difficile* cells in different media. Log kill represents the difference of log(CFU) between buffer-treated and enzyme-treated cells. Abbreviations: CFU (C), colony forming unit in no-enzyme control group; CFU (E), colony forming unit in enzyme-treated group; E, enzyme CD11; PBS, phosphate buffered saline; M, growth medium; BHI, brain-heart infusion; YE, yeast extract; P, peptone; P3, peptone No. 3; D, dextrose; C, L-cysteine; LB, Lysogeny Broth. Results are the means ± standard deviations of triplicate assays.

We have previously identified CD11 to be an effective lytic enzyme with *N*-acetylmuramoyl-L-alanine amidase activity against vegetative cells of *C. difficile* at mid-log phase^32^. To test the lytic activity of CD11, the killing assay was performed in both PBS and growth medium. As shown in Fig. 1a, CD11 reduced cell count (expressed as colony forming units, CFU) by 3–4 log units in PBS; however, the enzyme lost all activity in *C. difficile* growth medium.

*C. difficile* growth medium is a rich broth containing peptides, amino acids, and carbohydrates, many of which are not well defined. To investigate whether any component of growth medium inhibited the activity of CD11, a cell wall based spectrophotometric assay was performed in the presence of different medium components. The isolated cell wall fragments of *C. difficile* were susceptible to CD11 in solutions containing all the nutrients tested, although the digestion occurred at different rates, to different extents, and in some cases, with noticeable lag periods (Fig. 1b). This result suggests that the activity of CD11 was indeed influenced by nutrients, but none of the nutrients completely inhibited enzyme activity. When CD11 was tested against cell wall fragments in growth medium, which contained all the nutrients, the activity and the digestion rate were surprisingly much higher than those observed in PBS (Fig. 1b), while this enzyme was essentially inactive against live cells in growth medium (Fig. 1a).

The intact cell wall in its natural surroundings is in close contact with other molecules and structures of the cells, such as membrane proteins, teichoic acids, and lipids. However, isolated cell wall materials consist of a fragmented peptidoglycan structure, and many of the associated proteins, lipids, and other molecules are removed during preparation by SDS treatment and boiling. As a consequence, the peptidoglycan in isolated cell wall fragments is highly exposed during enzymatic digestion.

To better understand how the intact cell wall interacts with CD11 in various nutrients, enzyme activity on *C. difficile* cells was measured using a plating assay. CD11 showed enhanced activity in the presence of dextrose compared with PBS treatment (Fig. 1c), consistent with the result of the cell wall based assay. However, in all the other components tested, the activity of CD11 decreased relative to that in PBS, which was contrary to the observations in the cell wall assay (Fig. 1b). The most dramatic decrease in CD11 activity in the plating assay was observed in the presence of growth medium and brain-heart infusion (BHI), where enzyme activity was essentially abolished.

Besides the influence of medium components on enzyme activity, Fig. 1c also shows the effect of nutrients alone on cell viability. In yeast extract, peptone, peptone No. 3 and dextrose, the CFUs were comparable to that in PBS, and CD11 was highly active in the presence of these nutrients. Cell viability was low in L-cysteine (23% of that in PBS), even in the absence of enzyme. While we have not elucidated the cause of low cell viability, CD11 retained full activity in L-cysteine with close to 4-log cell killing. In the presence of growth medium or BHI, cell growth occurred (CFU almost doubled than that in PBS) during the 3 h incubation at room temperature. Cells did not grow in PBS or simple media (like yeast extract, peptone, or dextrose) without supplementation of all the essential nutrients necessary for metabolism^6^, and the final CFU reflected the initial inoculum size. In growth medium and BHI, with all essential nutrients present, cells could grow, albeit slowly at room temperature, leading to elevated cell count compared to PBS treatment. Collectively, these results suggest that CD11 is inactive in the presence of nutrients that support cell growth, e.g., resulting in metabolically competent cells.

To further verify this hypothesis, diluted LB medium (which is much less rich than BHI) and diluted growth medium were used to study the activity of CD11 and the behavior of cells. As shown in Fig. 1d, CD11 was able to reduce cell viability by 60% and 97% in 50% LB and 20% LB, respectively. When cells were incubated in 20% growth medium in the absence of enzyme, the CFU increased by 70% (0.24 log units difference) compared to PBS; cell viability was now reduced by ~30% in the presence of CD11. However, in 50% growth medium, the cell count doubled without enzyme (0.33 log units higher than in PBS), while only ~5% killing was observed in the presence of CD11. Collectively, these results suggest that cells were resistant to CD11 when their growth and metabolism were largely maintained, and CD11 was most active when cells were metabolically dormant.

### Binding behavior of CD11 to cells and cell wall materials in the presence of medium nutrients

**Figure 2:**
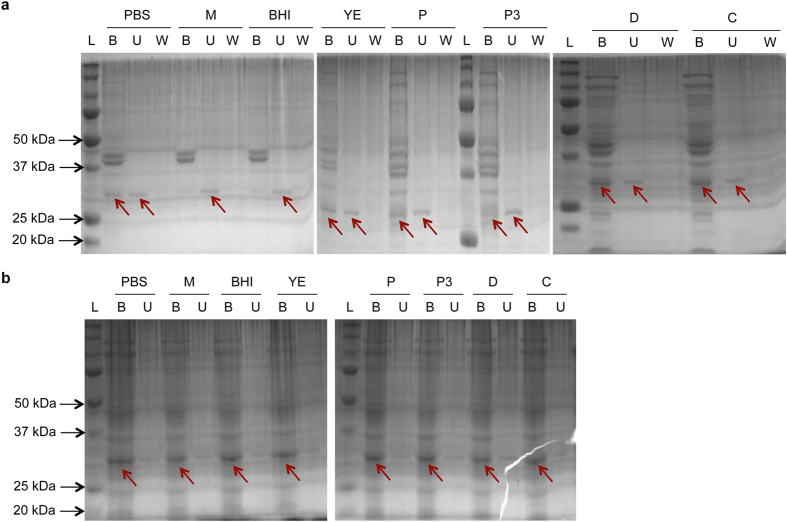
Binding of CD11 to *C. difficile* cell wall under different nutrient conditions. (**a**) Binding of CD11 to intact cell wall in live *C. difficile* cells. (**b**) Binding of CD11 to isolated cell wall materials. Abbreviations: PBS, phosphate buffered saline; M, growth medium; BHI, brain heart infusion; YE, yeast extract; P, peptone; P3, peptone No. 3; D, dextrose; C, L-cysteine; B, bound fraction (pellet); U, unbound fraction (supernatant); W, wash, loosely bound fraction; L, molecular weight ladder. The arrows indicate the band of CD11.

As described above, CD11 was not inhibited by the growth medium, and the resistance of *C. difficile* cells to CD11 in growth medium was not a result of medium-induced enzyme inactivation. Given the complexity of the cell wall, it is possible that the cell wall substrate for CD11 may not be easily accessible in metabolically competent cells in growth medium. To test this hypothesis, a pull-down assay was performed with the bound enzyme detected by SDS-PAGE^33^,^34^; this assay was facilitated by the solubility of CD11 in the conditions tested (Supplementary Fig. S1). When CD11 was incubated with mid-log phase cells under different nutrient conditions, the enzyme was found in both the pellet fraction and the supernatant fraction in PBS, yeast extract, peptone, peptone No. 3, dextrose, and L-cysteine (Fig. 2a). CD11 in the supernatant fraction was either the free enzyme unbound to the cells, or the enzyme associated with degraded and solubilized cell wall. Due to the solubility and stability of the enzyme, the detection of enzyme in the pellet fraction was a consequence of CD11 binding to the cells. In addition, the observed binding was specific, as the enzyme did not bind *Bacillus anthracis* (a phylogenetically close relative of *C. difficile*) cells in either PBS or BHI (Supplementary Fig. S1). In growth medium and BHI, however, the enzyme was only observed in the supernatant fraction, indicating that there was no or significantly reduced enzyme binding to cells in these media.

To assess whether the inability of CD11 to bind cells in growth medium and BHI was related to substrate accessibility, the pull-down assay was performed using isolated cell wall materials as the substrate. As shown in Fig. 2b, CD11 was observed in both the bound and unbound fractions under all conditions tested, although most of the enzyme was bound to the insoluble substrate. The presence of multiple protein bands besides CD11 in the bound fraction indicates that the isolated cell wall was not pure peptidoglycan, and some cellular proteins were not removed by SDS and boiling treatment.

Collectively, these results suggest that the enzyme activity was dependent on efficient substrate binding, and that the poor activity of CD11 in growth media was a result of reduced cell wall accessibility rather than altered structure of the cell wall peptidoglycan. This further suggests that certain components on the cell surface (such as lipids and teichoic acids) underwent a change in conformation and/or physicochemical property when cells were transferred from PBS to BHI or growth medium, which caused the peptidoglycan to be less accessible to CD11.

### Exploring the effect of teichoic acids on enzyme binding and cell killing

**Figure 3:**
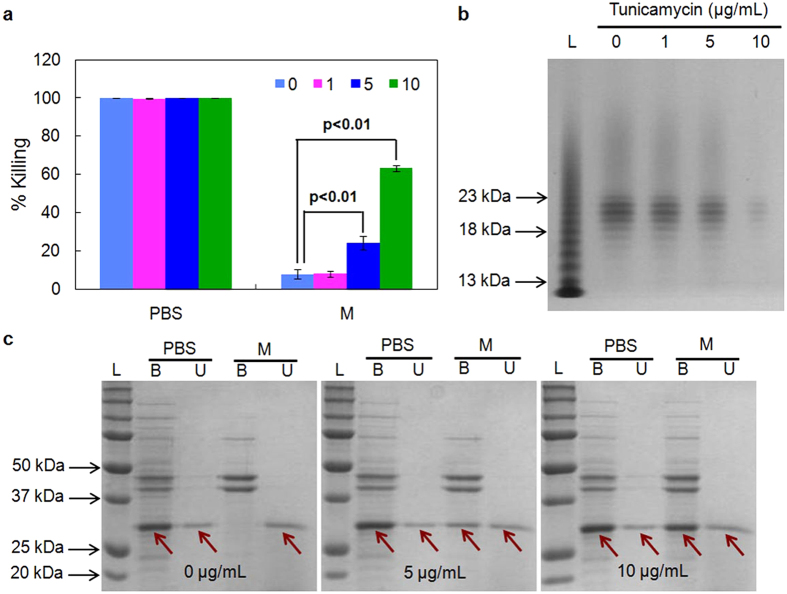
Effect of tunicamycin on activity and cell binding of CD11. (**a**) Activity of CD11 against mid-log phase *C. difficile* cells cultured in different concentrations of tunicamycin. (**b**) The level of isolated WTAs from *C. difficile* cells treated with tunicamycin. (**c**) Binding of CD11 to mid-log phase, tunicamycin-treated *C. difficile* cells in growth medium. Abbreviations: PBS, phosphate buffered saline; M, growth medium; B, bound fraction (pellet); U, unbound fraction (supernatant); L, molecular weight ladder. Arrows indicate CD11. Data represents means ± standard deviations of triplicate experiments, and the p value is calculated by a two-tailed student *t*-test (p value = 0.004 and 0.00002 for 5 and 10 μg/mL tunicamycin, respectively).

The cell walls of many Gram-positive bacteria are decorated with anionic glycopolymers termed wall teichoic acids (WTAs). WTAs consist of phosphodiester-linked polyol repeat units, and are covalently attached to cell wall peptidoglycan^35^,^36^. WTAs play important roles in drug resistance^35^,^37^,^38^,^39^, and have been shown to restrict access of endolysins to the cell wall in *Listeria monocytogenes* in PBS - Tween 20^40^. In addition, PlyG and PlyL (*Bacillus* phage endolysins) show higher activity against *B. anthracis* cells when the S-layer polymers (including teichoic acids and teichuronic acids) are removed^41^. To understand whether WTAs also regulate the exposure of *C. difficile* cell wall to CD11, cells were cultured in the presence of tunicamycin, an antibiotic that inhibits the first step of WTA biosynthesis^36^,^42^,^43^,^44^, and were assayed for enzyme activity. As shown in Fig. 3a, tunicamycin had little effect on enzyme activity in PBS and on cell viability within the range of concentrations tested (Supplementary Fig. S4); however, in growth medium, cells treated with tunicamycin became sensitive to CD11, and the cell killing activity of CD11 increased more than 10-fold in growth medium with 10 μg/mL tunicamycin.

To confirm that tunicamycin inhibited WTA synthesis in *C. difficile*, WTAs were isolated from *C. difficile* cells grown to the same OD at different concentrations of tunicamycin. Figure 3b shows that the level of WTAs decreased with increasing dose of tunicamycin. With fewer WTAs present at the cell surface, enhanced cell binding was observed for CD11 in growth medium, while the binding in PBS was not influenced (Fig. 3c). These results indicate that the level of WTAs in *C. difficile* contributed to the accessibility of cell wall to CD11 in growth media, and higher enzyme activity was accompanied by better substrate binding.

### Contribution of CD11 domains to cell binding and lysis

**Figure 4:**
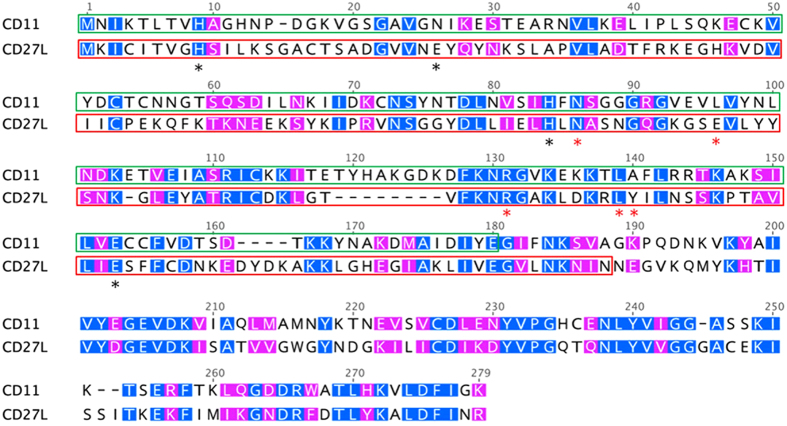
Sequence alignment of CD11 and CD27L. Amino acid residues are numbered based on their positions in the alignment (including gaps). Identical residues are white on a blue background. Residues with similar properties throughout are white on a pink background. The catalytic domains of CD11 and CD27L are labeled in green and red boxes, respectively. The unboxed regions are the binding domains. The active sites of CD27L are highlighted with black asterisks, while the hydrogen bonding sites important for CD27L activity are marked with red asterisks.

The majority of lytic enzymes are modular proteins, with at least one catalytic domain and one cell wall binding domain^2^,^6^. In CD27L, the N-terminal 179 amino acid residues belong to the catalytic domain while the remaining 91 amino acid residues are the binding domain^31^,^45^. In the catalytic domain of CD27L, the active site comprises of H9, E26, H84, and E144, which are essential for zinc binding, while N86, E96, R122, L130, Y131 are involved in hydrogen bonding and are thought to directly contribute to the enzyme activity^31^. The L98 residue in CD27L is conserved among other lysins and are usually occupied by a hydrophobic amino acid, with L, V, I, F, Y and W reported for this position, and the L98W mutation in CD27L or CD27L_1–179_ does not reduce the enzyme activity^31^. CD11 showed 57% similarity to CD27L with the NCBI BLASTp algorithm, and it contains most of these conserved residues (H9, H83, E143, N85, R130, L138, L97) (Fig. 4). Based on sequence alignment, the N-terminal 179 amino acid residues in CD11 were taken as the putative catalytic domain (corresponding to the N-terminal 175 residues in CD27L), while residues 170–271 were treated as the putative binding domain. The additional 10-residues (170–179) were included in the putative binding domain to help ensure completeness and proper folding of this domain.

**Figure 5:**
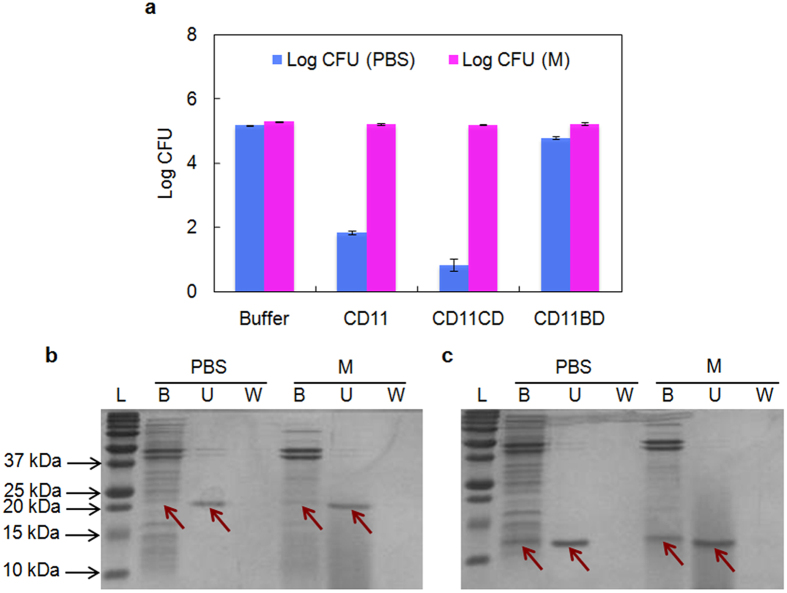
The lytic activity and cell binding capability of separate domains of CD11. (**a**) The activity of the catalytic domain and binding domain of CD11 (CD11CD and CD11BD, respectively) against mid-log phase *C. difficile* cells in PBS and growth medium. (**b,c**) Binding of CD11CD and CD11BD respectively to mid-log phase *C. difficile* cells. Abbreviations: PBS, phosphate buffered saline; M, growth medium; B, bound fraction (pellet); U, unbound fraction (supernatant); W, wash, loosely bound fraction; L, molecular weight ladder. Data represents means ± standard deviations of triplicate assays, and the highlighted band is CD11.

To understand to what extent the independent domains contribute to cell binding and lysis in different nutrient conditions, the catalytic and binding domains of CD11 (CD11CD and CD11BD, respectively) were expressed and purified separately (Supplementary Fig. S2), and tested against vegetative *C. difficile* cells harvested from mid-log phase. In PBS, CD11CD was more active than full-length CD11 with 1-log higher cell killing, while CD11BD failed to kill a significant number of cells (Fig. 5a). These results are consistent with those previously reported for CD27L, where the catalytic domain CD27L_1–179_ was more potent at lysing live cells and cell saculi in PBS than full-length CD27L, while the binding domain CD27L_180–270_ was inactive^31^. In growth medium, however, both CD11CD and CD11BD were inactive, as was the case for full-length CD11. Unlike the full-length CD11, which could bind to cells only in PBS, the pull-down results indicated that CD11CD interacted with cells weakly in both growth medium and PBS (Fig. 5b) while CD11BD was bound to cells in both PBS and growth medium (Fig. 5c).

## Discussion

Lysins recently have received attention as potential alternatives to conventional antibiotics, and have been explored for medical applications in animals and humans. However, there remains a lack of information on the enzymology of lysins in growth-supporting media, which hinders their application in relevant therapeutic settings. In the present study, we have shown that the resistance of *C. difficile* cells to CD11 in growth medium is attributed to the lack of enzyme binding to the cell surface, which is likely caused by some degree of physical interference by WTAs on the exterior surface of the bacterial cell wall. To the best of our knowledge, this is the first report of the possible explanation for the inactivation of lytic enzymes in growth media.

WTAs are important components of the Gram-positive bacterial cell envelope, and have long been investigated for their role in mediating bacterial pathogenesis, antimicrobial resistance, and cell division. WTAs of *S. aureus* limit recognition of innate immune receptors in Drosophila^44^ and are essential for nasal colonization in humans^46^, thus facilitating infection. WTAs in *L. monocytogenes* restricted access of the cell wall binding domains of Ply118, Ply511, and PlyP40 to the peptidoglycan and lack of these polymers enabled unrestricted access of the cell wall binding domains to the cell wall surface^40^. The binding domain of the endolysin Lyb5 and the autolysin AcmA also showed enhanced peptidoglycan binding upon removal of teichoic acids with trichloroacetic acid^47^,^48^.

**Figure 6:**
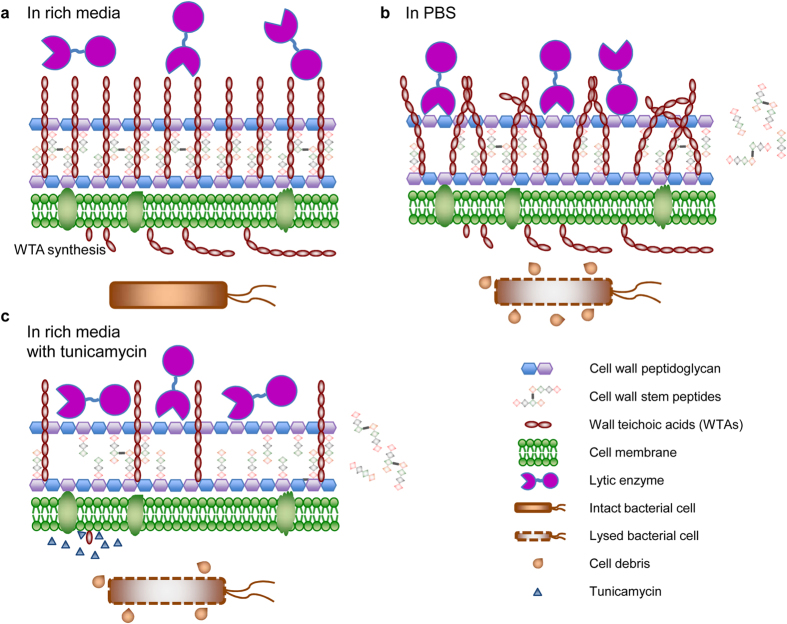
Schematic demonstration of a possible mechanism of medium-induced inactivation of CD11 against *C. difficile*. (**a**) The stabilization of wall teichoic acids (WTAs) in growth medium leads to a lack of cell binding or cell wall cleavage by CD11, and the cells are not killed by CD11. (**b**) The conformational change of WTAs in PBS allows for the access of CD11 to the cell wall, the digestion of the stem peptides from the peptidoglycan, and the lysis of *C. difficile* cells. (**c**) Partial removal of WTAs by tunicamycin increases the accessibility of the cell wall to CD11 in growth medium, and leads to cell wall degradation and cell lysis by CD11.

WTAs were present at similar levels in cells suspended in both growth medium and PBS (Supplementary Fig. S3). Moreover, WTAs are unlikely to renew during the short incubation period in the pull-down assay given their complicated structure and multi-step biosynthesis. Thus, the observed enzyme binding in PBS and the lack of binding in growth medium and BHI in the absence of tunicamycin is likely not due to a change in amount of WTAs but possibly due to a change in its conformation (Fig. 6). The conformation of polyelectrolytes such as WTA is known to be sensitive to the composition of the medium. Moreover, factors such as salt concentration, pH, and temperature have been shown to affect the composition of WTAs (e.g., the content of D-alanyl esters^49^), which in turn has been shown to influence WTA conformation and the binding of proteins to the cell wall^50^ as well as bacterial resistance to antimicrobial peptides and enzymes^49^,^51^. A proposed explanation for our results is that WTAs have a rigid conformation in rich media that restricts access of lytic enzymes to the peptidoglycan layer. In PBS, WTAs assume a random and flexible conformation^50^,^52^,^53^, facilitating close contact of large molecules with the peptidoglycan layer. We further note that the smaller proteins CD11CD and CD11BD could bind intact cells in growth medium, while the larger enzyme CD11 could not, consistent with a role for steric hindrance. Indeed, it has been shown that the S-layer polymers of *B. anthracis* act like molecular sieves and favor the smaller catalytic domain of PlyG instead of the full-length enzyme^41^. Consistent with this hypothesis, partial elimination of WTA biosynthesis using tunicamycin restores CD11 binding and activity in growth medium. As a possible future direction, CD11 may be applied in combination with a WTA-degrading enzyme (such as phosphatase) to achieve efficient *in vitro* eradication of *C. difficile* in rich media.

The interior part of bacterial cell walls is different from the exterior part because of distinct microenvironments and the attachment of different moieties such as WTAs, lipoteichoic acids, and lipids. In their native environment, autolysins and endolysins both work from the inside. When applied externally, although they are still highly active, the activity can be influenced by many external and internal factors. VALs, functioning at the initial stage of phage infection, naturally act on vigorously growing cells from the outside, and are expected to be active in growth medium^3^. Indeed, fusion of the M23 endopeptidase domain of the VAL Orf 73 to the binding domain of the endolysin Lys170, which is inactive in rich media, generates a chimeric enzyme with strong lytic and killing effect in rich growth media^3^. In future work, a similar approach may be adopted for the engineering of CD11 by identifying VALs against *C. difficile* and forming chimeras through domain swapping.

Cell lytic enzymes usually do not target bacterial spores directly because of the protective spore coat and the structural differences between the spore cortex and the vegetative cell wall. However, lytic enzymes have been shown to be active against spores that are germinating or when used in combination with germinants or other enzymes^54^,^55^. CD11, as a potent lytic enzyme against *C. difficile*, may also find use in eradicating *C. difficile* spores in combination with reagents that induce germination.

The interaction chemistry between lytic enzymes and metabolically active bacterial cells remains a new and unexploited area for microbiologists and enzyme engineers. More exploration is needed to achieve a complete and comprehensive understanding of the cross-talk between enzymes and live cells, with the final goal of the development of lysin-dependent effective methods to eradicate pathogenic bacteria. Our work provides a new perspective on the engineering of cellular interactions with lytic enzymes, and will facilitate the development of *in vivo* therapeutics targeting the human gut and blood where there is an abundance of nutrients.

## Methods

### Plasmid construction and cloning

The *cd11* gene was synthesized with codons optimized for *Escherichia coli* expression, and was cloned into the plasmid pGS21a between *Nde* I and *Xho* I restriction sites with a C-terminal His-tag sequence (Genscript, NJ). The recombinant plasmid pGS21a-CD11 was transformed into *E. coli* BL21 (DE3) Star competent cells (Invitrogen) and sequenced. The catalytic domain of CD11 (*cd11cd*) was PCR-amplified with the forward primer 5′-gcggcatatggctaacatcaaaacgc-3′ and the reverse primer 5′-ccgcctcgaggttgaaaataccttcgtag-3′, and the binding domain of CD11 (*cd11bd*) was PCR-amplified with the forward primer 5′-gcggcatatggctattgacatctacgaagg-3′ and the reverse primer 5′-ccgcctcgagtttgccaatgaaatcc-3′ (the underlined letters indicate the restriction sites). Both genes were cloned into pGS21a (pGS21a-CD11CD and pGS21a-CD11BD, respectively), sequenced, transformed into BL21 (DE3) Star competent cells and sequenced again. The cells harboring the correct plasmid were stored as glycerol stocks (8%, v/v, glycerol) at −80 °C.

### Protein expression and purification

Fifty milliliters of LB containing 100 μg/mL ampicillin were inoculated with 10 μL of the BL21 (DE3) Star glycerol stock harboring pGS21a-CD11, pGS21a-CD11CD, or pGS21a-CD11BD, and incubated at 37 °C overnight with 220 rpm shaking. The overnight culture was transferred to 250 mL fresh LB medium supplemented with 100 μg/mL of ampicillin with a 50-fold dilution factor, and the culture was incubated at 37 °C with 220 rpm shaking until OD_600_ reached around 0.6 (~3 h). IPTG was added to a final concentration of 0.5 mM to induce protein expression, and the culture was incubated at room temperature with 185 rpm shaking for 4 h. Cells were centrifuged at 4,000 rpm for 20 min at 4 °C, resuspended in 30 mL of native purification buffer (50 mM sodium phosphate, pH 8, 500 mM NaCl), aliquoted into two tubes, and frozen at −80 °C.

One tube of cells was thawed at room temperature, mixed with 0.1 mg of DNase (Sigma) and 75 μL of phenylmethanesulfonyl fluoride (PMSF, 0.1 M solution in ethanol, Sigma), and sonicated at 60% power with 3 s pulses for 4 min on ice with 3 s intervals (Sonics Vibra-Cell). The cell lysate was centrifuged at 4,000 rpm for 20 min at 4 °C, and the supernatant was incubated with 1 mL high-density Ni-NTA (Gold Biotechnology) pre-equilibrated with native purification buffer in a 20 mL purification tube (Bio-Rad) with 120 rpm shaking at 4 °C. The flow-through was discarded, and the column was washed with 60 resin volumes of native wash buffer (50 mM sodium phosphate, pH 8, 500 mM NaCl, 20 mM imidazole). The overexpressed protein was eluted with 6 resin volumes of native elution buffer (50 mM sodium phosphate, pH 8, 500 mM NaCl, 250 mM imidazole), and dialyzed against 50 mM potassium phosphate buffer (pH 7) with a dilution factor of 100,000. The dialyzed protein was filter sterilized and stored at 4 °C.

### Medium and bacterial cell culture

*Clostridium difficile* strain 630 was purchased from ATCC (BAA-1382-FZ). The growth medium contained 38 g/L Brain-Heart Infusion (BHI, BD), 5 g/L yeast extract (Sigma), and 0.1% L-cysteine (Sigma). *C. difficile* cells were grown at 37 °C without shaking in an anaerobic chamber (Coy Laboratories). BHI contains 15 g/L peptone, 10 g/L peptone No. 3, 2 g/L dextrose, 5 g/L NaCl, 2.5 g/L Na_2_HPO_4_. For the preparation of agar plates, 38 g BHI, 5 g yeast extract and 15 g agar (Sigma) were mixed in 1 L DI water, autoclaved at 121 °C for 20 min, cooled to ~50 °C, and supplemented with 10 mL of filter-sterilized 10% (w/v) L-cysteine. Around 30 mL of the mixture was poured into each sterile 100 × 15 mm petri dish (Krackeler). The plates were kept in the anaerobic chamber for at least one day prior to use.

### Enzyme activity against live cells

A single colony of *C. difficile* cells was used to inoculate 3 mL growth medium. After overnight growth at 37 °C, 100 μL culture was transferred to 3 mL fresh medium and incubated at 37 °C until OD_600_ reached ~0.5. Two milliliters of cell suspension were centrifuged at 13,000 rpm for 2 min at room temperature, washed once with sterile phosphate buffered saline (PBS), pH 7.4, and the cell pellet was resuspended in 2.5 mL PBS. Then, 250 μL of 50 mM potassium phosphate buffer, pH 7 (control) or 250 μL of 0.3 mg/mL enzyme (CD11, CD11CD, or CD11BD) in 50 mM potassium phosphate buffer, pH 7 was mixed with 250 μL of PBS, growth medium, 38 g/L BHI, 5 g/L yeast extract, 15 g/L peptone, 10 g/L peptone No. 3, 2 g/L dextrose, or 0.1% L-cysteine in 1.5-mL sterile tubes. Forty microliters of cells were added to each tube, and incubated at room temperature for 3.5 h. The suspension was serially diluted, and 35 μL of the undiluted and the diluted cell suspensions were spread on agar plates. The plates were incubated at 37 °C for 20 h, and the number of colonies was counted. All steps were performed in the anaerobic chamber, and all the buffers and nutrients were sterilized and kept in the chamber for at least one day before use.

To study the effect of nutrient concentration, different percentages of Lysogeny Broth (LB) and the growth medium were tested similarly. LB or growth medium (250 μL) was mixed with 250 μL of either potassium phosphate buffer (50 mM, pH 7) or CD11. Since the media were diluted 1:1 with buffer, the concentrations of nutrients were only half of those in the pure media, and these media were called 50% media (i.e., 50% LB or 50% growth medium). Likewise, 20% media were prepared by mixing 100 μL of media with 150 μL of sterile DI water and 250 μL of either potassium phosphate buffer or CD11. Cell suspension (40 μL) was added to each mixture, incubated, diluted, and plated.

### Isolation of cell wall peptidoglycan fragments

The cell wall peptidoglycan of *C. difficile* was isolated according to a previously reported method^56^. Briefly, *C. difficile* cells were grown in growth medium at 37 °C overnight, and transferred to 200 mL fresh medium at a dilution factor of 30. The culture was incubated at 37 °C until OD_600_ reached ~0.5. The culture was aliquoted into 50 mL tubes, removed from the anaerobic chamber, centrifuged at 4000 rpm for 20 min at 4 °C, and introduced into the chamber again. The medium was discarded, and the pellet was suspended in 50 mL sterile PBS. The cell suspension was removed from the chamber, centrifuged at 4,000 rpm for 20 min at 4 °C, and introduced into the chamber. The supernatant was discarded, and the pellet from 50 mL culture was resuspended in 0.5 mL of 4% SDS. The cell-SDS mixture was removed from the chamber, boiled at 95 °C for 30 min, and incubated at room temperature for 16 h with 200 rpm shaking. The suspension was boiled again at 95 °C for 10 min, cooled to room temperature, and centrifuged at 15,000 rpm for 15 min at room temperature. The pellet was resuspended in 1 mL of DI water and centrifuged at 15,000 rpm for 2 min at room temperature. This step was repeated 15 times to remove SDS, and the pellet was resuspended in 0.5 mL of DI water. The washed cell wall peptidoglycan fragments were stored at 4 °C.

### Activity of CD11 against cell wall peptidoglycan in different nutrients (OD assay)

Fifty microliters of *C. difficile* cell wall peptidoglycan fragments were mixed with 450 μL of PBS, growth medium, 38 g/L BHI, 5 g/L yeast extract, 15 g/L peptone, 10 g/L peptone No. 3, 2 g/L dextrose, or 0.1% L-cysteine in 1-mL cuvettes, and then supplemented with 300 μL of PBS. CD11 (100 μL of 0.3 mg/mL in 50 mM potassium phosphate buffer, pH 7) was added to the mixture immediately before measurement. As a control, 50 μL of cell wall peptidoglycan fragments were mixed with 750 μL of PBS and 100 μL of 50 mM potassium phosphate buffer (pH 7). The cuvettes were placed in a spectrophotometer and the change in OD_600_ was monitored every 5 s for 5 min.

### Cell-based pull-down assay

*C. difficile* cells were grown overnight and sub-cultured into 6 mL fresh medium with a dilution factor of 30. The culture was incubated at 37 °C until OD_600_ reached ~0.5. Cells were centrifuged at 13,000 rpm for 2 min at room temperature, washed once with PBS, and resuspended in 1 mL PBS. Potassium phosphate buffer (50 mM, pH 7, 120 μL) was mixed with 250 μL of PBS, growth medium, 38 g/L BHI, 5 g/L yeast extract, 15 g/L peptone, 10 g/L peptone No. 3, 2 g/L dextrose, or 0.1% L-cysteine. CD11, or its separate domains, at 0.3 mg/mL was supplemented to 30 μL, incubated for 5 min at room temperature, and 100 μL of cell suspension was added. The mixtures were incubated for 1 min at room temperature, and centrifuged at 13,000 rpm for 2 min at room temperature. The supernatants were transferred to fresh tubes for gel analysis. The pellets were resuspended in 500 μL PBS, vortexed for 5 s, and centrifuged at 13,000 rpm for 2 min at room temperature. The supernatants and the pellets were separated and stored for sodium dodecyl sulfate-polyacrylamide gel electrophoresis (SDS-PAGE) analysis.

As a control to check the precipitation of CD11 in different nutrients, the enzyme was mixed with the same ratios of nutrients and buffers, except that the 100 μL of cell suspension was replaced by 100 μL of PBS. The mixtures were incubated at room temperature for 1 min, and 50 μL was collected from each tube for gel analysis. The remaining mixtures were centrifuged at 13,000 rpm for 2 min at room temperature. Although there was no visible pellet in any tube; nonetheless, 50 μL of the upper portion of the supernatant was collected for SDS-PAGE analysis.

Liquid samples (50 μL each) were diluted 1:1 with 2X Laemmli buffer (Bio-Rad) supplemented with beta-mercaptoethanol (Sigma) at 5% final concentration, while the pellets were suspended in 50 μL of the Laemmli buffer containing beta-mercaptoethanol. All the samples were boiled at 95 °C for 10 min, cooled to room temperature, and loaded into 10% or 15% denaturing polyacrylamide gels.

### Cell wall-based pull-down assay

Seventy microliters of 50 mM potassium phosphate buffer (pH 7) were mixed with 20 μL of 0.4 mg/mL CD11 and 150 μL of PBS, growth medium, 38 g/L BHI, 5 g/L yeast extract, 15 g/L peptone, 10 g/L peptone No. 3, 2 g/L dextrose, or 0.1% L-cysteine. The mixtures were incubated at room temperature for 5 min, and mixed with 60 μL of isolated cell wall peptidoglycan fragments. After 1 min incubation at room temperature, the mixtures were centrifuged at 13,000 rpm for 2 min at room temperature, and the supernatants were transferred to fresh tubes for gel analysis. The pellets were resuspended in 300 μL PBS, vortexed for 5 s, and centrifuged again. The supernatants and the pellets were separated and stored for gel analysis. For SDS-PAGE analysis, all the samples were prepared in the same way as described above.

### Inhibition of wall teichoic acids by tunicamycin

*C. difficile* cells were grown overnight, sub-cultured into 10 mL fresh medium supplemented with 10 μL DMSO (BDH Chemicals), 9 μL DMSO + 1 μL tunicamycin (10 mg/mL stock solution in DMSO, Alfa Aesar), 5 μL DMSO + 5 μL tunicamycin, or 10 μL tunicamycin, and grown at 37 °C until OD_600_ reached 0.5. Cells were then harvested for binding assay or plating assay.

### Isolation and gel electrophoresis of wall teichoic acids

The isolation and polyacrylamide gel electrophoresis (PAGE) of WTAs was similar to a previously reported protocol^57^. Briefly, overnight culture of *C. difficile* was transferred to 50 mL fresh medium (with or without tunicamycin) with a 30-fold dilution factor and incubated at 37 °C until OD_600_ reached 0.5. Cells were centrifuged at 4,000 rpm for 15 min at 4 °C, washed once with 50 mM Tris-HCl (pH 8), resuspended in 20 mL of 4% (w/v) SDS in 50 mM Tris-HCl (pH 8), and boiled at 95 °C for 1 h. The cell sacculi were centrifuged at 14,000 *g* for 10 min at room temperature, washed once with 2% (w/v) NaCl in 50 mM Tris-HCl (pH 8) and five times with 50 mM Tris-HCl (pH 8), and digested with 4 mL of 0.1 mg/mL Protease K (Sigma) in 20 mM Tris-HCl (pH 8) containing 0.5% (w/v) SDS at 50 °C for 4 h. The digestion product was spun at 14,000 *g* for 10 min at room temperature, washed once with 2% (w/v) NaCl in 50 mM Tris-HCl (pH 8) and seven times with DI water, mixed with 120 μL of 0.1 M NaOH, and incubated at room temperature for 16 h with 120 rpm shaking. After incubation, the mixture was centrifuged at 14,000 *g* for 10 min at room temperature, and the supernatant containing isolated WTAs was stored at 4 °C.

Extracted WTAs were analyzed on 20% polyacrylamide gels by loading 20 μL of samples mixed with 5 μL of loading buffer (50% glycerol, 0.1 M Tris, 0.1 M Tricine, pH 8.2, supplemented with bromophenol blue). Gels were developed in Tris-glycine running buffer (25 mM Tris, 192 mM glycine) using constant voltage (150 V) for 2.5 h, washed with DI water, stained with 1% alcian blue, and destained with DI water.

## Additional Information

**How to cite this article**: Wu, X. *et al*. Wall Teichoic Acids Are Involved in the Medium-Induced Loss of Function of the Autolysin CD11 against *Clostridium difficile. Sci. Rep.* **6**, 35616; doi: 10.1038/srep35616 (2016).

## Supplementary Material

Supplementary Information
