# Supplementary material for: Wall Teichoic Acids Are Involved in the Medium-Induced Loss of Function of the Autolysin CD11 against Clostridium difficile
Source: Sci Rep. 2016 Oct 19;6:35616. doi: 10.1038/srep35616 (PMC5069495; doi:10.1038/srep35616)
Supplement: Supplementary Information [file srep35616-s1.pdf]

Supplementary Information

Wall Teichoic Acids Are Involved in the Medium-Induced Loss of Function of the Autolysin CD11 against *Clostridium difficile*

Xia Wu[1, 2], Elena E. Paskaleva[1], Krunal K. Mehta[1, 2], Jonathan S. Dordick[1, 2*], Ravi S. Kane[3*]

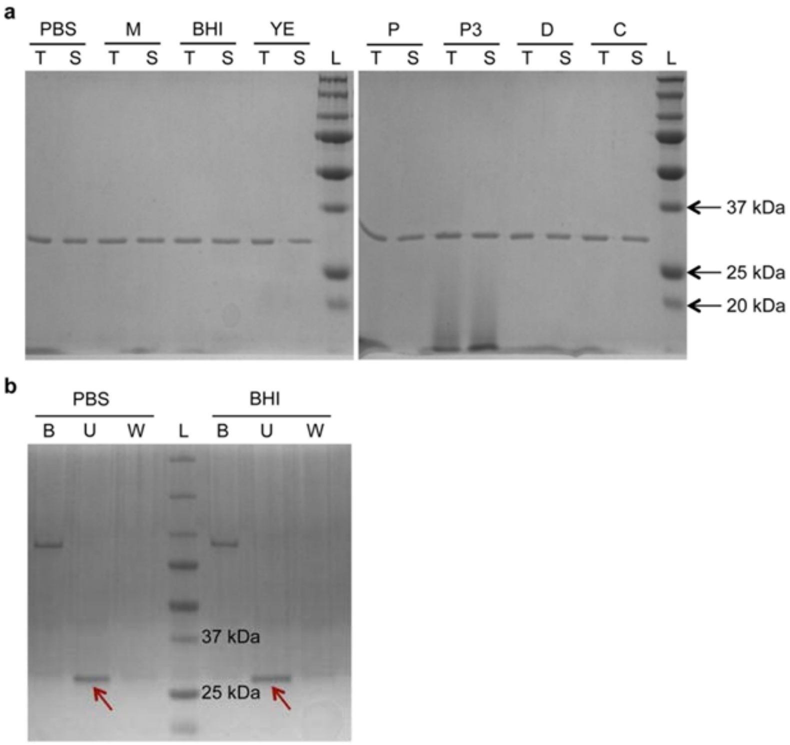

Fig. S1. The pull-down study of CD11 in different nutrients and in the presence of *B. anthracis* cells. (a). The solubility and stability of CD11 in different nutrients. **(b).** Measurement of non-specific adsorption of CD11 to *B. anthracis* (a phylogenetically close relative of *C. difficile*) cells by the pull-down assay. Abbreviations: PBS, phosphate buffered saline; M, growth medium; BHI, brain heart infusion; YE, yeast extract; P, peptone; P3, peptone No. 3; D, dextrose; C, L-cysteine; T, total mixture; S, soluble fraction of the mixture; B, bound fraction (pellet); U, unbound fraction (supernatant); W, wash; L, molecular weight ladder. The highlighted band is CD11.

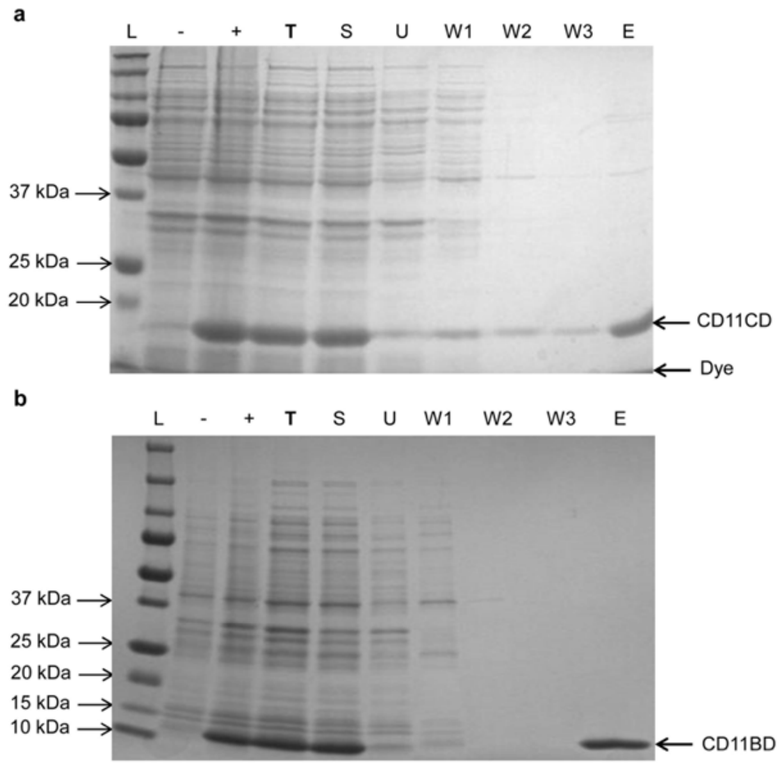

Fig. S2. SDS-PAGE analysis of the expression and Ni-NTA purification of the catalytic domain and the binding domain of CD11. (a). Expression and purification of the catalytic domain of CD11 (CD11CD). **(b).** Expression and purification of the binding domain of CD11 (CD11BD). Abbreviations: L, molecular weight ladder; -, whole cells before induction; +, whole cells after induction; T, total proteins (total cell lysate); S, soluble proteins (soluble cell lysate); U, unbound fraction during purification, proteins that did not bind to Ni-NTA column; W1, W2, and W3, flow-through from the first, second, and third column wash; E, elution, proteins eluted from the Ni-NTA column.

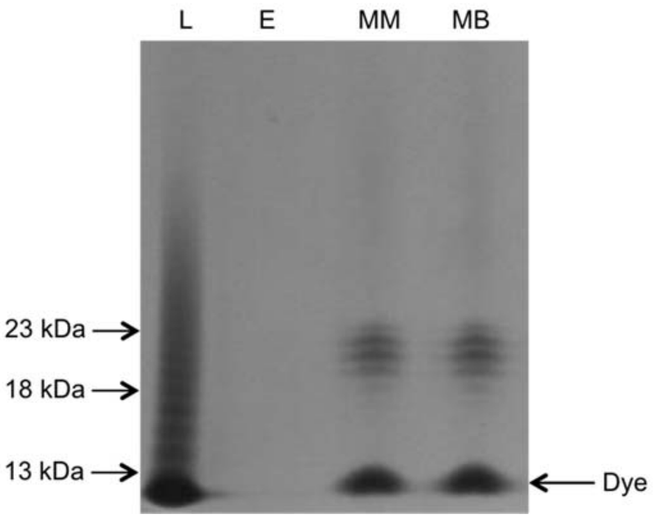

Fig. S3. Polyacrylamide gel electrophoretic analysis of wall teichoic acids isolated from differentially treated *C. difficile* cells. Cells sub-cultured from overnight growth were grown till OD_{600}=~0.5. After harvesting and washing, cells were resuspended in the same amount of fresh growth medium or phosphate buffered saline, and incubated at room temperature for 3 h, followed by isolation of wall teichoic acids. Abbreviations: L, ladder; E, empty well; MM, cells transferred from growth medium to fresh growth medium; MB, cells transferred f rom growth medium to phosphate buffered saline.

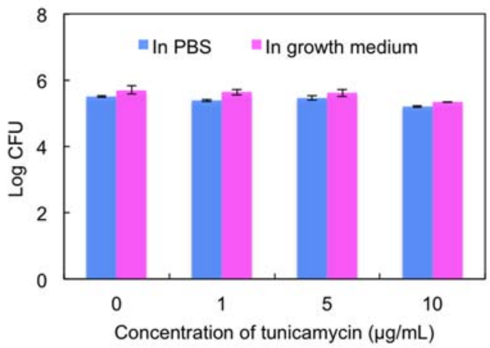

Fig. S4. Viability of C. difficile cells in PBS and growth medium in the presence of tunicamycin. Cells sub-cultured from overnight growth were supplemented with different concentrations of tunicamycin and grown for 3 h. After harvesting and washing, cells were serial-diluted and spread on agar plates for overnight growth at 37 °C, and colony forming units were counted. Abbreviations: PBS, phosphate buffered saline. Data represents means ± standard deviations of triplicate assays.
